# Surgical Management of a Giant Mediastinal Schwannoma Associated with Neurofibromatosis Type 1: A Case Report

**DOI:** 10.70352/scrj.cr.25-0150

**Published:** 2025-08-22

**Authors:** Shin-nosuke Watanabe, Daisuke Kimura, Kengo Tani, Takahiro Sasaki, Shuta Kimura, Chisaki Muto, Tsubasa Kato, Masahito Minakawa

**Affiliations:** Department of Thoracic and Cardiovascular Surgery, Hirosaki University, Graduate School of Medicine, Hirosaki, Aomori, Japan

**Keywords:** neurofibromatosis, schwannoma, mediastinal tumor, posterolateral thoracotomy

## Abstract

**INTRODUCTION:**

Neurogenic tumors commonly develop in the posterior mediastinum in both pediatric and adult patients. In patients with neurofibromatosis type 1, distinguishing benign schwannomas from malignant peripheral nerve sheath tumors is challenging. In this study, we aimed to present the surgical management of a giant schwannoma that required differentiation from a malignant peripheral nerve sheath tumor.

**CASE PRESENTATION:**

A 15-year-old boy presented with a large right mediastinal mass discovered on chest radiography at high school admission. Subsequent contrast-enhanced CT of the chest showed the development of a large tumor (16.0 × 12.5 × 11.8 cm) occupying approximately two-thirds of the right thoracic cavity, with atelectasis of the lower lobe of the right lung. The patient was histopathologically diagnosed with a benign schwannoma associated with neurofibromatosis type 1 through a thoracoscopic biopsy of the tumor and had received oral selumetinib at 50–70 mg/day for 11 months. Surgical excision was performed because of tumor progression and suspected malignant transformation. Right posterolateral thoracotomy with the opening of the 6th intercostal space was performed by extending the anterior skin incision along the abdominal rectus muscle and separating the 6th costal cartilage and diaphragmatic margin along the chest wall. The tumor was completely removed by resecting numerous adhesions between the tumor and the surrounding tissues and coagulating several nutrient vessels that flowed into the tumor, while resecting the lower lobe of the lung. The postoperative course was uneventful. The pathological examination revealed no malignancy. Subsequent contrast-enhanced CT of the chest revealed no residual tumors.

**CONCLUSIONS:**

Posterolateral thoracotomy with the separation of the costal cartilage and diaphragmatic margin along the chest wall could achieve safe surgery for a giant mediastinal schwannoma.

## Abbreviations

CE-CT
: contrast-enhanced CT
FDG/PET
: fluorine-18 fluoro-2-deoxy-d-glucose PET
H3K27
: me3 histone H3 lysine 27 trimethylation
MEK
: mitogen-activated protein kinase kinase
MMS
: mediastinal mass syndrome
MPNST
: malignant peripheral nerve sheath tumor
NF-1
: neurofibromatosis type 1
PNs
: plexiform neurofibromas
SOX-10
: SRY-related HMG-BOX gene 10
VATS
: video-assisted thoracic surgery

## INTRODUCTION

Neurogenic tumors most commonly develop in the posterior mediastinum in both pediatric and adult patients. They include schwannomas, neurofibromas, and MPNST, the latter of which can be life-threatening in patients with NF-1. Differentiating neurogenic tumors is challenging.^1,2)^ In this study, we aim to present the surgical management of a patient with a giant schwannoma that developed in the mediastinum associated with NF-1 and required differentiation from MPNST, along with a clinical literature review.

## CASE PRESENTATION

**Fig. 1:**
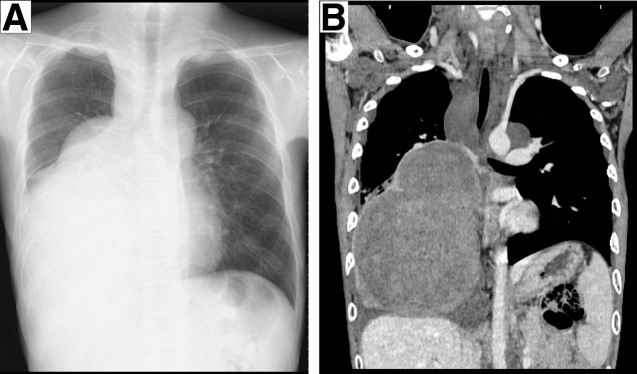
Imaging findings at the first visit. (**A**) The chest radiograph shows wide areas of consolidation in the right thoracic cavity. (**B**) Chest CT shows the development of a large tumor occupying the right middle and lower thoracic cavities, with atelectasis of the lower lobe of the right lung.

**Fig. 2:**
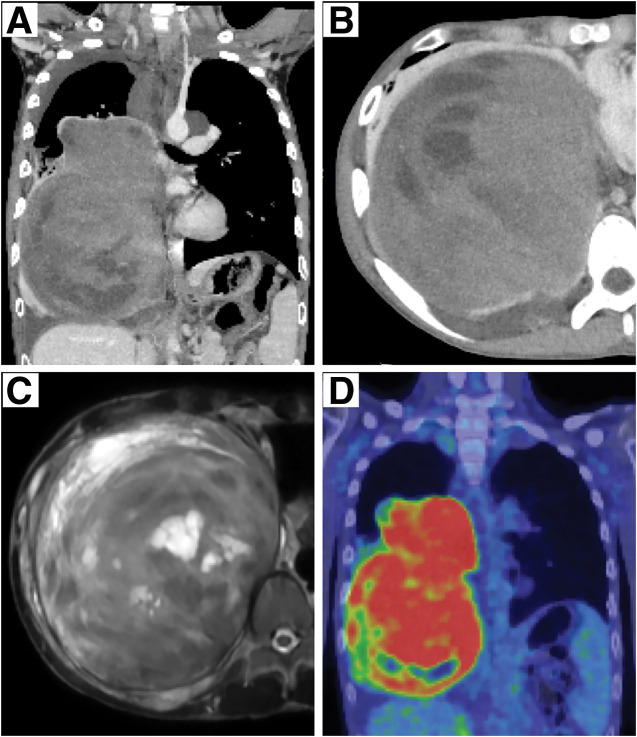
Preoperative imaging findings. (**A**, **B**) Chest CT showing tumor progression with mediastinal compression and pleural effusion. (**C**) MRI showed no invasion of the surrounding tissues or the vertebral canal. (**D**) The tumor showed high uptake on fluorine-18 fluoro-2-deoxy-d-glucose PET.

A 15-year-old boy presented with a large right mediastinal mass that was incidentally discovered on chest radiography at high school admission (**Fig. 1A**). Subsequent CE-CT of the chest showed the development of a large tumor measuring 16.0 × 12.5 × 11.8 cm, occupying the right middle and lower thoracic cavity, with atelectasis of the lower lobe of the right lung (**Fig. 1B**). The patient exhibited no symptoms such as dyspnea or chest pain and was diagnosed with NF-1 because of multiple café-au-lait spots on physical examination inherited from his mother. Spirometry was performed, revealing restrictive ventilatory impairment: vital capacity (VC) was 1550 mL (%VC, 44.3%), and forced expiratory volume in 1 s (FEV1) was 1090 mL (%FEV1, 72.7%). He underwent a thoracoscopic biopsy of the large tumor and was histopathologically diagnosed with a benign schwannoma that was immunohistochemically positive for S100 protein, H3K27me3, and SOX-10, and negative for an MIB-1 index of <5%. He received oral selumetinib at 50–70 mg/day at the discretion of a pediatrician with careful monitoring. After 11 months, the patient experienced pitting edema and proteinuria, which are adverse events of selumetinib, and complained of back pain that required the administration of narcotic analgesics. CE-CT and MRI concurrently showed the progression of the tumor measuring 19.5 × 14.7 × 12.6 cm, with mediastinal compression and pleural effusion, but without invasion of the surrounding tissues or extension into the vertebral canal (**Fig. 2A**–**2C**). Surgical excision was decided because the tumor showed a maximum standardized uptake value of 8.2 on FDG/PET–CT, indicating potential malignant transformation without evidence of metastatic disease (e.g., in the lung or bone) (**Fig. 2D**).

**Fig. 3:**
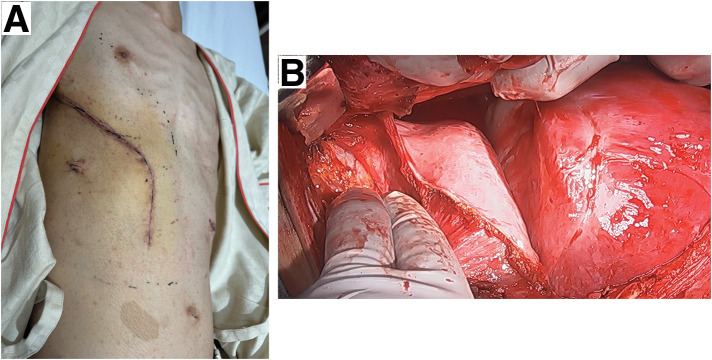
Surgical approach of the present case. (**A**) Skin incision performed during surgery. (**B**) Right posterolateral thoracotomy with the opening of the 6th intercostal space and the separation of the 6th costal cartilage and diaphragmatic margin along the chest wall without opening the peritoneum.

**Fig. 4:**
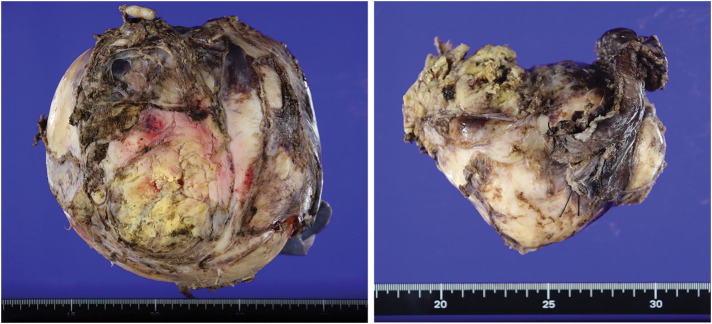
Formalin-fixed resected tumor comprising 2 pieces that weighed 2015 g.

An approach that allowed for a sufficient field of view for complete tumor resection was considered. We planned to perform a right posterolateral thoracotomy and extend the anterior skin incision along the abdominal rectus muscle (**Fig. 3A**). The chest was opened at the 6th intercostal space. The 6th costal cartilage was separated, and the diaphragmatic margin was separated along the chest wall without opening the peritoneum (**Fig. 3B**). The tumor occupied approximately two-thirds of the right thoracic cavity, was covered with the mediastinal pleura, and was densely adherent to the ribs, thoracic vertebrae within the mediastinum, and the lower lobe of the right lung with pleural effusion. The tumor was a capsule-covered lesion that did not invade the surrounding tissues, including the chest wall, diaphragm, or pericardium. No enlarged lymph nodes were observed in the mediastinum. Meticulous dissection was performed to separate the tumor from numerous adhesions to the ribs and thoracic vertebrae. Several nutrient vessels, which had formed from the intercostal arteriovenous system and azygos veins to the tumor, were coagulated. We could not identify the origin of the tumor because of its extensive adhesion to the ribs and thoracic vertebrae. The tumor comprised 2 pieces. First, we resected the lower part of the tumor, followed by the upper part. Subsequently, we resected the lower lobe of the right lung, which had been destroyed because of long-term compression by the upper part of the tumor. The resected tumor weighed 2015 g (**Fig. 4**). The operation lasted for 6 h and 23 min. The total amount of blood loss was 3370 g.

The postoperative course was uneventful. The patient was discharged 6 days after surgery without significant postoperative complications and did not require the narcotic analgesics that were preoperatively administered for back pain. Baseline CE-CT, performed approximately 3 weeks after surgery, showed no residual tumors.

**Fig. 5:**
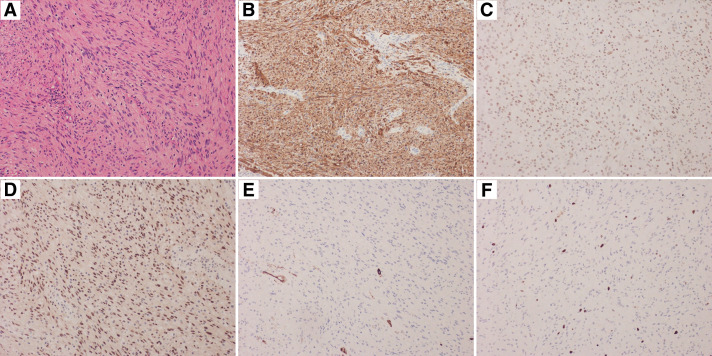
Pathological findings of the resected tumor. (**A**) Hematoxylin–eosin staining revealed spindle-shaped cells with eosinophilic capsules and oblong nuclei that proliferated fascicularly and densely, and partially enlarged atypia of the nuclei of these cells (×200). Immunohistochemical staining revealed that the tumor cells were positive for (**B**) S100 protein, (**C**) H3K27me3, and (**D**) SOX10, and negative for (**E**) CD34 and (**F**) MIB-1 index of 5% (×200). CD, cluster of differentiation; H3K27me3, me3 histone H3 lysine 27 trimethylation; SOX10, SRY-related HMG-BOX gene 10

Pathological examination revealed spindle-shaped cells with eosinophilic capsules and oblong nuclei that proliferated fascicularly and densely. Similarly, the nuclei showed partial enlargement and atypia, without hemorrhage or necrosis (**Fig. 5A**). Immunohistochemical staining revealed that the tumor cells were positive for S100 protein, H3K27me3, and SOX-10 but negative for CD34 and MIB-1 index of 5%, indicating no malignancy (**Fig. 5B**–**5F**). There were no pathological differences between the 2 resected pieces. On cytological examination, the intraoperative pleural effusion contained only mesothelial cells, histiocytes, neutrophils, and lymphocytes, indicating no malignancy.

## DISCUSSION

NF-1, also known as von Recklinghausen’s disease, is an autosomal dominant inherited disorder that causes various lesions in multiple organs, including the skin and the nervous, ocular, and skeletal systems. NF-1 is caused by heterozygous pathogenic variants of the *NF1* tumor suppressor gene on chromosome 17q11.2, resulting in the constitutive activation of the RAS pathway via MEK, which is implicated in cell proliferation and promotes tumor growth and progression.^3,4)^ PNs, which are histologically benign nerve sheath tumors, are the most frequent tumors associated with NF-1. However, many cases, including superficial PNs, are not detectable on physical examination, and imaging modalities such as CT and MRI are essential for detecting internal PNs.^1,5)^ PNs develop early in childhood, growing most rapidly (>20% increase yearly) during this period, whereas the tumor grows slowly or is suppressed in older adolescents and adults.^6,7)^ Schwannoma is a major PN type that most likely develops in the posterior mediastinum. The incidence of schwannoma in patients with NF-1 is unknown, and there are no sex-related differences in the incidence. Almost all posterior mediastinal schwannomas develop in a paravertebral location from the sympathetic ganglia or intercostal nerves^8)^ and usually cause no symptoms unless there is a secondary extrinsic compression of the corresponding structures, such as the trachea or esophagus. Symptoms of compression, such as chest and back pain, dyspnea, dysphagia, Pancoast syndrome, and Bernard–Horner syndrome, may occur when the tumor becomes large.^9–12)^

Although the optimum therapeutic option for a symptomatic PN is surgical removal, this approach is technically challenging and frequently infeasible owing to the tumor’s location or size, and it is associated with a high risk of postoperative complications. A comprehensive retrospective study revealed that complete tumor removal was successful in only 15% of cases, while 43% of patients who underwent surgery experienced PN recurrence.^13)^ In 2020, selumetinib, an oral selective MEK1/2 inhibitor, was approved by the United States Food and Drug Administration for the treatment of NF-1-associated symptomatic inoperable PNs in children older than 2 years.^14)^ Han et al.^15)^ systematically evaluated the efficacy and safety of selumetinib in patients with NF-1. Ten clinical trials involving 268 patients revealed that the pooled objective response rate was 68.0% (95% confidence interval [CI], 58.0%–77.3%), and the disease control rate was 96.8% (95% CI, 90.8%–99.7%).

However, clinicians should reconsider the indications for surgery in cases of PNs associated with NF-1 that are discontinued before medical treatment because of disease progression, adverse events, or suspected malignant transformation (e.g., MPNST). MPNST is a rare and highly aggressive cancer that originates from Schwann cells and constitutes approximately 10% of all soft tissue sarcomas. The cumulative lifetime risk of developing MPNST is 0.001% in the general population and 8%–13% in patients associated with NF-1.^16,17)^ Notably, in the areas of existing internal PNs, the risk of developing MPNST is 20-fold higher.^18)^ Patients with MPNST are usually symptomatic not due to compression but rather owing to the invasion of the corresponding structures and may present with respiratory and/or gastrointestinal symptoms. Therefore, progressive severe pain in patients with schwannoma warrants clinical suspicion of potential malignant transformation. While Martin et al. systematically reviewed the diagnostic accuracy of MRI and PET/CT in differentiating between benign schwannomas and MPNST, several studies have reported that some cases of schwannomas that were diagnosed as malignant owing to increased FDG uptake on PET/CT were finally diagnosed as benign based on the Ki-67 index.^19–21)^ These findings indicate that the preoperative differentiation of benign from malignant schwannomas through PET/CT usually requires validation through pathological examination.

Surgical removal is the only curative treatment option for patients with suspected MPNST because radiation therapy and conventional chemotherapy did not improve overall survival.^22–24)^ Regarding the perioperative management of giant mediastinal tumors, careful consideration should be given to the risk of acute circulatory collapse and respiratory failure due to MMS during induction of anesthesia. MMS occurs more frequently, particularly in giant anterior or superior mediastinal tumors during surgery in the supine or lateral decubitus position than in giant posterior mediastinal tumors because the thoracic vertebrae may help prevent the compression of surrounding organs, including the trachea, main bronchus, and great vessels, even in the lateral decubitus position.^25–27)^ Therefore, the surgical resection of the giant posterior mediastinal tumors is generally considered safe under general anesthesia, intubation with a double-lumen endotracheal tube, and patient placement in the lateral decubitus position. Regarding the surgical approach for giant posterior mediastinal tumors, a posterolateral thoracotomy is preferred to secure a sufficient field of vision.^12)^ Chen et al.^11)^ reported that VATS could be successfully performed for small tumors measuring <8 cm without conversion to open thoracotomy, whereas open thoracotomy might be more suitable for tumors that are larger, suspected to be malignant, or located at the costophrenic angle or thoracic apex. Moreover, dumbbell-shaped tumors, which extend toward the spinal cord, require high posterolateral or posterior transaxillary thoracotomy before costotransversectomy and laminectomy, whereas tumors that develop at the cervicothoracic junction require a combined approach using VATS and a supraclavicular incision.^11,12)^ In our case, we decided to separate the 6th costal cartilage and the diaphragmatic margin along the chest wall owing to the invasion of the tumor into the diaphragm and the need for resection of the lower lobe of the right lung; however, we did not consider an S-shaped skin incision along the costal arch or opening of the peritoneum. Our primary intraoperative challenge was in controlling the amount of bleeding from several nutrient vessels to the tumor and oozing while reducing numerous adhesions between the tumor and the parietal pleura of the posterior mediastinum. Open surgery for hemostasis of the hemothorax may be challenging owing to the fragile nature of the vascular tissue in NF-1.^28,29)^ Therefore, meticulous surgical technique is required when dissecting the tumor and managing the associated vasculature.

## CONCLUSIONS

We present a case of a giant mediastinal schwannoma in NF-1 that required differentiation from an MPNST. Clinicians should reconsider the indications for surgery in cases of schwannomas associated with NF-1 that are discontinued before medical treatment. Adequate surgical vision can be secured through posterolateral thoracotomy by separating the costal cartilage and diaphragmatic margin along the chest wall in the lateral decubitus position.
